# Acute Imidacloprid Exposure Alters Mitochondrial Function in Bumblebee Flight Muscle and Brain

**DOI:** 10.3389/finsc.2021.765179

**Published:** 2021-12-01

**Authors:** Chloe Sargent, Brad Ebanks, Ian C. W. Hardy, T. G. Emyr Davies, Lisa Chakrabarti, Reinhard Stöger

**Affiliations:** ^1^School of Biosciences, Sutton Bonington Campus, University of Nottingham, Loughborough, United Kingdom; ^2^School of Veterinary Medicine and Science, Sutton Bonington Campus, University of Nottingham, Loughborough, United Kingdom; ^3^Department of Agricultural Sciences, University of Helsinki, Helsinki, Finland; ^4^Department of Biointeractions and Crop Protection, Rothamsted Research, Harpenden, United Kingdom; ^5^Medical Research Council Versus Arthritis Centre for Musculoskeletal Ageing Research, Birmingham, United Kingdom

**Keywords:** *Bombus terrestris*, mitochondria, imidacloprid, oxidative phosphorylation, high-resolution respirometry

## Abstract

Mitochondria are intracellular organelles responsible for cellular respiration with one of their major roles in the production of energy in the form of ATP. Activities with increased energetic demand are especially dependent on efficient ATP production, hence sufficient mitochondrial function is fundamental. In bees, flight muscle and the brain have particularly high densities of mitochondria to facilitate the substantial ATP production required for flight activity and neuronal signalling. Neonicotinoids are systemic synthetic insecticides that are widely utilised against crop herbivores but have been reported to cause, by unknown mechanisms, mitochondrial dysfunction, decreasing cognitive function and flight activity among pollinating bees. Here we explore, using high-resolution respirometry, how the neonicotinoid imidacloprid may affect oxidative phosphorylation in the brain and flight muscle of the buff-tailed bumblebee, *Bombus terrestris*. We find that acute exposure increases routine oxygen consumption in the flight muscle of worker bees. This provides a candidate explanation for prior reports of early declines in flight activity following acute exposure. We further find that imidacloprid increases the maximum electron transport capacity in the brain, with a trend towards increased overall oxygen consumption. However, intra-individual variability is high, limiting the extent to which apparent effects of imidacloprid on brain mitochondria are shown conclusively. Overall, our results highlight the necessity to examine tissue-specific effects of imidacloprid on respiration and energy production.

## Introduction

Intensification of agriculture has driven a global increase in pesticide use and fragmentation of pollinator habitats, often leading to a sparsity of resources within the foraging range of bees (1, 2). This requires bees to fly greater distances, thus increasing the energy demand of foraging. At the cellular level, energy is produced in the form of adenosine triphosphate (ATP) *via* oxidative phosphorylation (OXPHOS) within mitochondria (3). During OXPHOS, ATP is synthesised *via* the electron transport chain located in the inner mitochondrial membrane; electrons are transported *via* a series of carriers and protons are pumped into the intermembrane space producing a proton gradient which drives ATP synthesis *via* ATP synthase (4). It has been reported that the rate of electron transfer between OXPHOS enzymes in bees are the highest measured in any animal (5).

Carbohydrate, predominately in the form of trehalose, is the main source of energy in the nervous system and flight muscles of bees, however tissue-specific differences have been shown (5, 6). In the brain, evidence of β-oxidation to metabolise fatty acids and contribute to the high energetic demands has been reported, however there is little evidence of fatty acids fuelling flight in the thoracic muscle (6). Furthermore, glycogen is stored in the flight muscles which may be utilised to extend flight duration (5). These differences reinforce the importance of looking at multiple tissue types when analysing the mitochondrial function in bees.

In bees and other hymenopterans, the brain and flight muscle have particularly high densities of mitochondria to facilitate the substantial energy demands of neuronal signalling and flight activity (3, 5, 7). There is increasing evidence of an association between brain mitochondrial OXPHOS and behaviour in bees, suggesting that alterations in brain mitochondrial function, such as those caused by neonicotinoids, could induce changes in behaviours, such as aggression (7). External factors that affect mitochondrial function may have the greatest impact within tissues with the highest energy demands.

Neonicotinoids are a widely used group of systemic insecticides which have been shown to affect pollinator learning, memory, homing, and flight capacity (8, 9). These compounds target nicotinic acetylcholine receptors (nAChRs); the most prevalent excitatory neurotransmitter in the central nervous system of insects (10, 11). Neonicotinoids impair mitochondrial function and structure in bumblebees and honeybees (12–14) and exposure to the neonicotinoid imidacloprid leads to premature flight exhaustion and altered cognitive performance (15, 16). This may be associated with a transient excitatory affect caused by the overstimulation of nAChRs by imidacloprid and potentially lead to a reduction in flight activity and foraging capacity (15). The decline in flight activity associated with pesticide exposure may be due to insufficient ATP production in the flight muscle leading to premature exhaustion and flight inactivity. As mitochondria are responsible for the majority of ATP production during insect flight, an imidacloprid-induced impairment of mitochondrial function may impact on flight performance and neuronal signalling (17).

Here we seek to determine if and how acute exposure to field-realistic doses of imidacloprid may affect mitochondrial function and OXPHOS in the bumblebee, *Bombus terrestris*. Using high-resolution respirometry we analyse mitochondrial respiration in the brain and flight muscle of female worker bees (18). We analyse the oxygen consumption of three different respiration states: (i) Routine—the rate at which OXPHOS occurs in cells in the physiological coupling state, where ATP synthesis is coupled with the electron transport chain (ii) LEAK—a non-phosphorylating resting state of uncoupled respiration by the inhibition of ATP synthase; hence, oxygen consumption is associated with proton leak through the inner mitochondrial membrane and not *via* ATP synthesis-linked respiration; and (iii) maximum electron transport capacity (ET capacity)—the maximum rate of the electron transport pathway when not coupled to ATP synthase and at an optimum concentration of uncoupler (18). By analysing these three states we aim to identify if and how imidacloprid may affect the OXPHOS system.

## Materials and Methods

### Bee Husbandry

Commercial queen-right *B. terrestris audax* colony were obtained from Biobest^®^ (Westerlo, Belgium) between November 2020 and June 2021, and maintained at 26°C and 33% relative humidity. We fed the colony and age-matched laboratory reared bees on 2.0M sucrose solution and pollen (purchased from Agralan, UK) provided *ad libitum*. To create cohorts of bees of a similar age, pupae were harvested and stored in a separate plastic container adjacent to the colony until eclosion. Once eclosed, bees were placed into cohorts of 1–3 bees and contained in plastic deli pots (115 × 75 mm) until used for high-resolution respirometry (HRR).

### Imidacloprid Treatment

Individual bees were taken from their cohort and placed in a queen marker tube where they were starved for 1 h to increase the likelihood of feeding on the diet subsequently provided. For the insecticide exposed bees, a 2.92 M sucrose solution containing 10 ppb imidacloprid was then used to feed the bees. This field-realistic concentration was selected as it has been previously reported in pollen and nectar, foraging bees, and inside colonies (15). For the control bees, feeding was with 2.92 M sucrose solution only. Bees were allowed to feed for 10 mins and the quantify consumed was recorded. Any bees which did not feed during this period were excluded from the experiment. Thirty minutes after the end of feeding, bees were cold anesthetised (flight muscle *n* = 4, and brain *n* = 7; for both control and IMD groups).

### Flight Muscle and Brain Tissue Dissection

Cold-anaesthetised bees were killed by decapitation. The thorax was photographed to later measure the inter-tegular distance (representing body size) for each bee using ImageJ software (32). The brain or a small section of flight muscle were removed, using a scalpel and forceps, and then individually weighed in 1.5 ml tubes. Individual bees representing biological replicates were used for each tissue type sample. Then 100 μl of MiR05 buffer (0.5 mM EGTA, 3 mM MgCl_2_, 60 mM Lactobionic acid, 20 mM Taurine, 10 mM KH_2_PO_4_, 20 mM HEPES, 110 mM D-Sucrose, 1g/L BSA, pH 7.1) per 1 mg of muscle, or one whole brain, was added and the tissue mechanically homogenised. Then 100 μl (1 mg) of muscle tissue homogenate and all the brain homogenate were transferred into a high-resolution respirometry chamber for analysis.

### High-Resolution Respirometry

**Figure 1:**
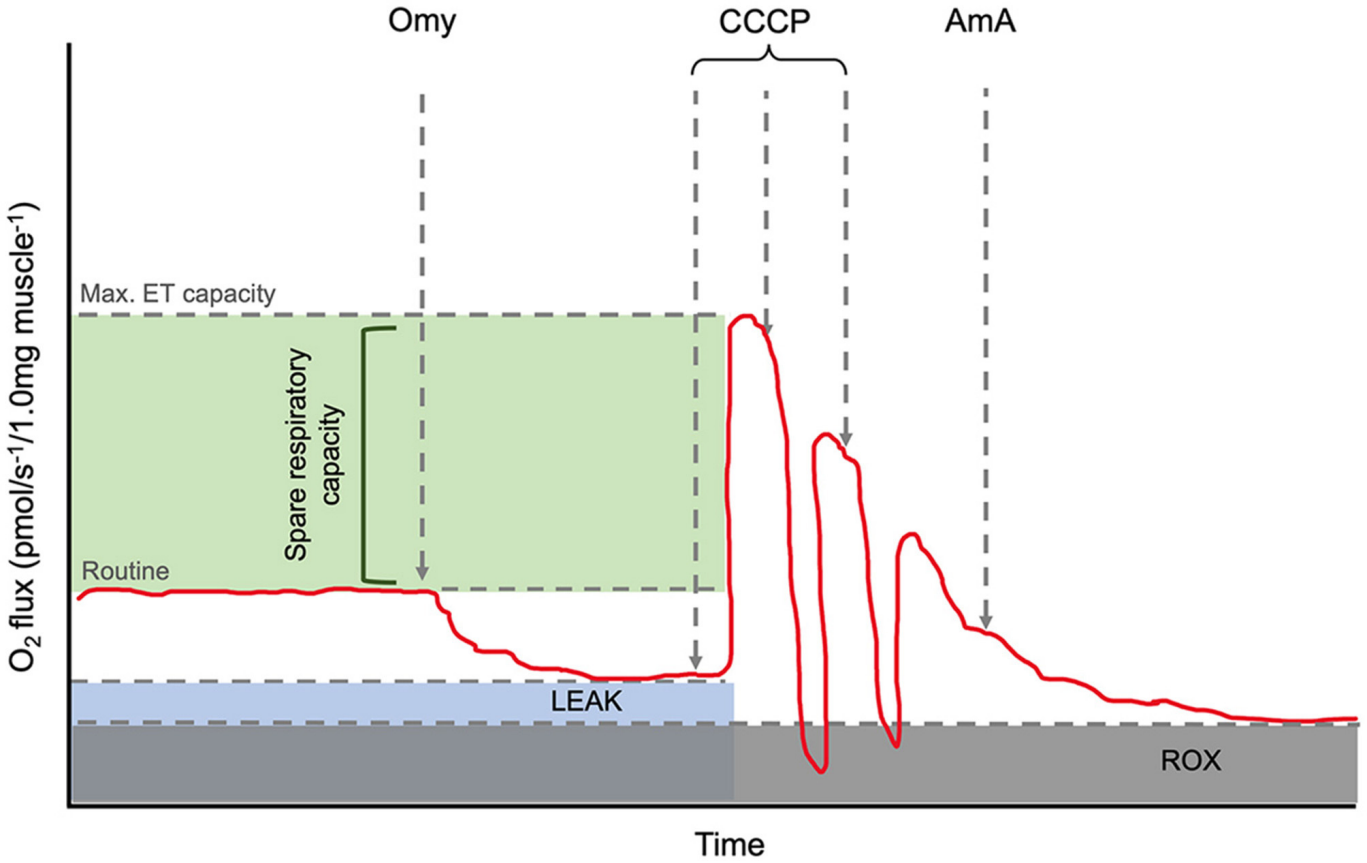
Annotated representation of the O_2_k oxygraph output. The red line is the oxygen flux. Routine is shown before the addition of Omy. LEAK state can be determined after the inhibition of ATP synthase by Omy. The maximum electron capacity is determined as the highest peak after CCCP titrations. The spare respiratory capacity and its relative value can be calculated *via* the calculation, [(ET capacity specific flux)/(Routine specific flux × 100)]. AmA, a complex III inhibitor, is added to determine the residual oxygen consumption (ROX) as the baseline state and allow for background correction. Abbreviations: Omy: oligomycin; CCCP: carbonyl cyanide m-chlorophenyl hydrazone; AmA: antimycin A.

High-resolution respirometry (HRR) was carried out using an Oroboros Oxygraph-2k (Oroboros^®^ Instruments, Innsbruck, Austria). The respirometer electrodes were calibrated daily to ensure oxygen concentration remained consistent for the duration of the experiment. All respiratory analyses were performed at 35°C as previous studies have reported this close to the average thoracic and head temperature of *B. terrestris* during flight (19, 20). Oxygen consumption of one brain and 1 mg flight muscle were analysed using the SUIT-003 protocol (18, 21). Routine respiration was measured followed by the addition of 1.0 μl of 5 μM oligomycin to determine LEAK state (inhibition of ATP synthase). Uncoupled maximal electron transport state was then measured by subsequent 10.0 μl titrations of 0.5 μM carbonyl cyanide m-chlorophenyl hydrazone (CCCP). Finally, 1 μl of 2.5 μM Antimycin A to determine the residual oxygen consumption (ROX) (Figure 1).

### Data Analysis

Raw data outputs were acquired using the O2k-Software (DatLab v7.4.0.4, Oroboros), for data acquisition and analysis to determine instrumental background corrected oxygen flux values. Data were subsequently analysed using R Team (22). Unpaired Student's *t*-tests were performed to determine whether there were differences between the mean oxygen flux at routine, LEAK and maximum ET capacity states, as well as the mean FCRs for routine and LEAK. The FCR can be defined as the ratio of oxygen flux in the different respiration states (routine and LEAK) that are normalised to obtain lower [residual oxygen consumption (ROX)] and upper (maximum ET capacity) limits of 0–1 (23). Each of these tests used adopted a Type I error rate of α = 0.5, with interpretations controlled for multiple comparisons as detailed in the supplementary materials. Oxygen consumption was also analysed in relation to the amount of sucrose consumed, bee age, and bee size (inter-tegular distance, ITD). There was no significant difference found in mean bee age or size between treatment groups in either tissue (Supplementary Table 2). However, as a range of ages check were used in both groups, one-way ANOVAs were used to check for potential age-related effects on mitochondrial function: age did not significantly affect any of the respiration states in the flight muscle or brain. To determine whether the amount of diet (sucrose solution with or without imidacloprid) consumed correlated with oxygen flux, Pearson's correlation coefficients were calculated; there was no effect in either the flight muscle or brain (Supplementary Table 3).

## Results

### Flight Muscle

**Figure 2:**
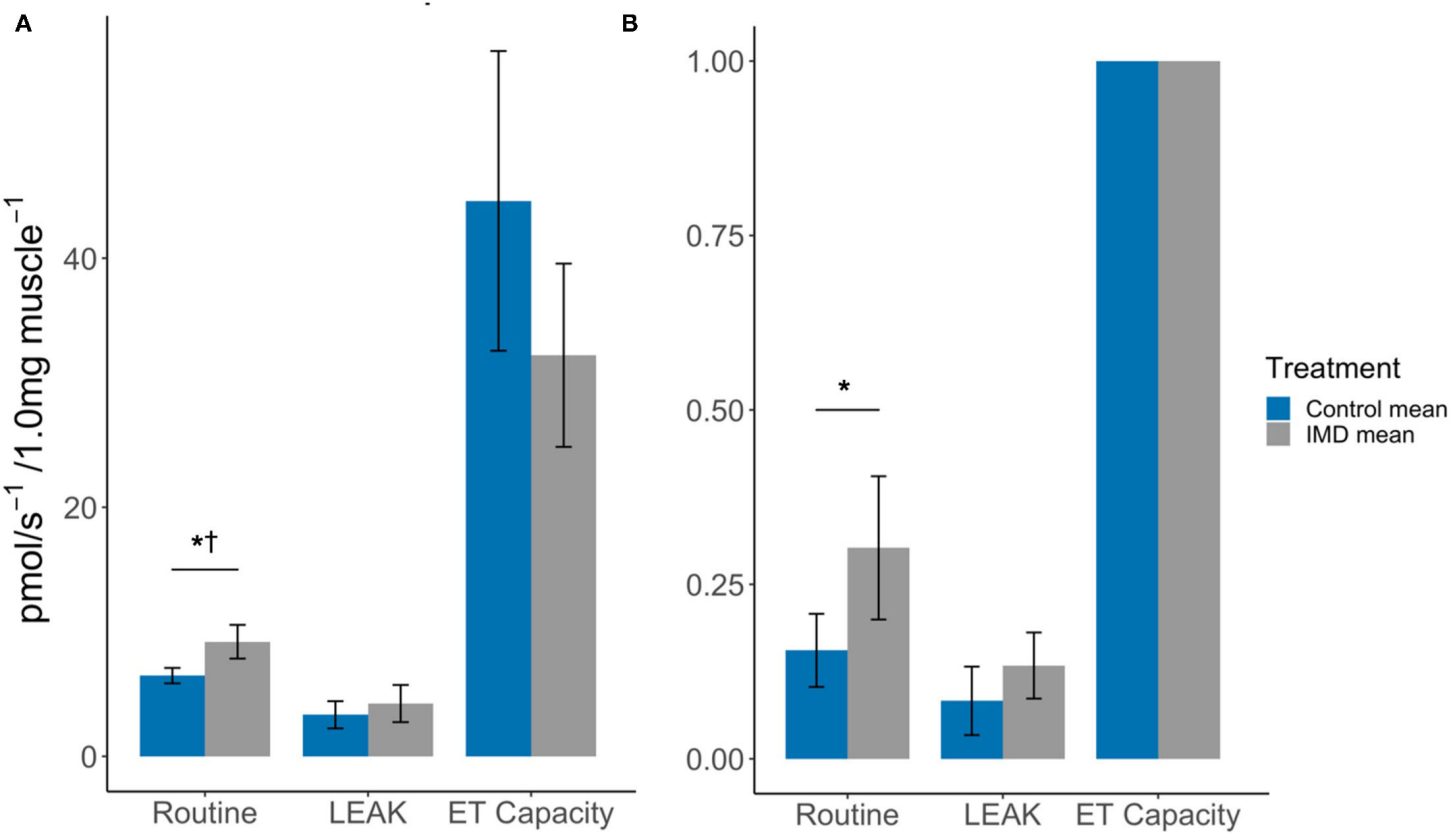
Effects of imidacloprid on flight muscle mitochondria at Routine, LEAK, and maximum ET capacity. States **(A)** Mean oxygen flux; and **(B)** Mean FCR, in flight muscle of imidacloprid and control worker bees. An unpaired students *t*-test was performed to determine significance between treatment groups. *p < 0.05. *p*-values marked with ^†^ were also significant after controlling for Type I error rates. Error bars show 95% confidence interval. *n* = 4 for each of the control and imidacloprid groups. Routine: routine respiration rate; LEAK: electron/H^+^ leak; ET capacity: maximum electron transport capacity.

Acute oral exposure of imidacloprid in worker bees significantly increased routine respiration in flight muscle (Oxygen flux: *t*_df = 6_ = −3.58, *p* = 0.012; FCR: *t*_df = 6_ = −2.50, *p* = 0.047, Figure 2; Table 1, note that the difference in FCR did not remain significant after correction for multiple comparisons, Supplementary Table 1). There were no significant differences found between the LEAK and maximum ET capacity states when comparing exposed and control bees (LEAK: oxygen flux, *t*_df = 6_ = −0.96, *p* = 0.376; FCR, *t*_df = 6_ = −1.45, *p* = 0.196. Maximum ET capacity: oxygen flux, *t*_df = 6_ = 1.72, *p* = 0.136, Figure 2; Table 1).

**Table 1:** Mean oxygen flux and flux control ratio values in the brain and flight muscle of imidacloprid and non-imidacloprid fed worker bees.

	**Flight Muscle**			**Brain**		
	**Control**	**IMD**	***p***	**Control**	**IMD**	***p***
	***n* = 4**	***n* = 4**		***n* = 7**	***n* = 7**	
**Routine**						
Oxygen flux						
Mean (s.e.)	6.48 (0.32)	9.20 (0.69)	0.012^*^ ^†^	2.75 (0.60)	7.23 (2.07)	0.060
Variance	0.40	1.91		2.54	30.02	
FCR						
Mean (s.e.)	0.16(0.03)	0.30(0.05)	0.047^*^	1.18 (0.38)	0.99 (0.13)	0.639
**LEAK**						
Oxygen flux						
Mean (s.e.)	3.33 (0.56)	4.24 (0.76)	0.376	1.62 (0.37)	4.82 (1.74)	0.097
Variance	1.24	2.32		0.97	21.24	
FCR						
Mean (s.e.)	0.08(0.03)	0.13(0.02)	0.196	0.73 (0.26)	0.60 (0.15)	0.67
**ET Capacity**						
Oxygen flux						
Mean (s.e.)	44.60 (6.14)	32.21 (3.75)	0.136	3.11 (0.59)	6.59 (1.12)	0.017^*^ ^†^
Variance	150.83	56.38		2.42	8.76	

*No flux control ratio (FCR) data are provided for the maximum electron transport capacity (ET capacity) as this is set to 1 to obtain the FCR for the routine and electron/H^+^ leak (LEAK) states. p-values were obtained from unpaired Student's t-tests, with significance*:

* *p < 0.05. p-values marked with*

† *were also significant after controlling for Type I error rates (Supplementary Table 1). IMD: imidacloprid; s.e.: ±1 standard error*.

The spare respiratory capacity (SRC) was also calculated for the flight muscle. The SRC describes the mitochondrial capacity to reach additional ATP demands that are greater than routine respiration levels and can be expressed as a quantitative value using the formula, SRC = [(ET capacity specific flux)/(Routine specific flux × 100)] (24) (Figure 1). Hence, the mean SRCs were 0.069 and 0.035 for the control and imidacloprid groups respectively. This may be explained by the increase in routine respiration but not the maximum ET capacity by imidacloprid which would result in a lower SRC.

### Brain

**Figure 3:**
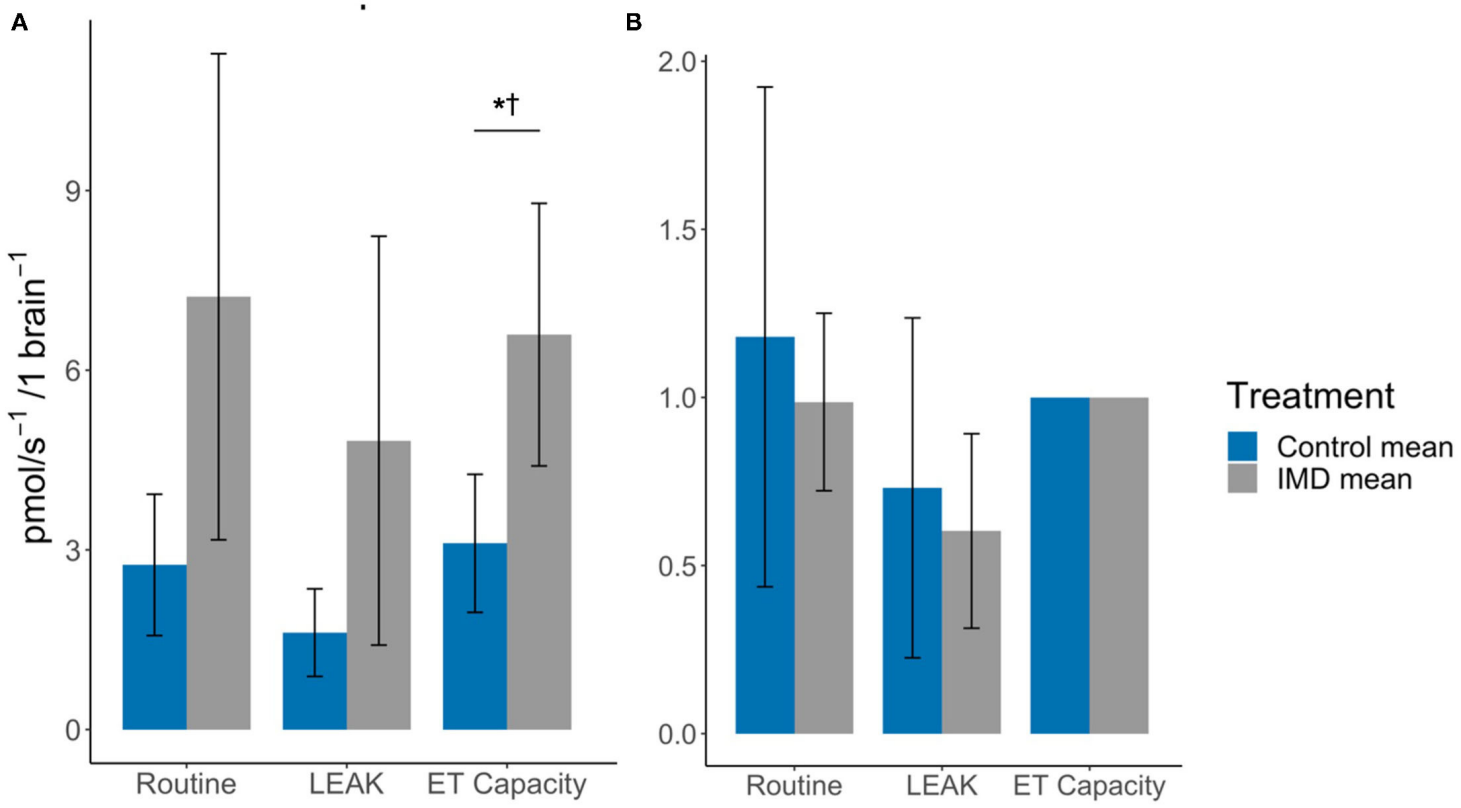
Effects of imidacloprid on brain mitochondria at Routine, LEAK, and maximum ET capacity. **(A)** Mean oxygen flux; and **(B)** Mean FCR, in the brains of imidacloprid and control worker bees. An unpaired students *t*-test was performed to determine significance between treatment groups *p < 0.05. *p*-values marked with ^†^ were also significant after controlling for Type I error rates. Error bars show 95% confidence interval. *n* = 7 for control and IMD groups. Routine: routine respiration rate; LEAK: electron/H^+^ leak; ET capacity: maximum electron transport capacity.

In brain tissue we found that the oxygen flux for imidacloprid fed bees had a significantly higher maximum ET capacity (*t*_df = 12_ = −2.76, *p* = 0.017), but not in Routine and LEAK (*t*_df = 12_ = −2.08, *p* = 0.060 and *t*_df = 12_ = −1.80, *p* = 0.097, respectively) (Figure 3; Table 1). However, there were no significant differences between the treatment groups in terms of FCRs (Routine: *t*_df = 12_ = 0.48, *p* = 0.639; LEAK: *t*_df = 12_ = 0.43, *p* = 0.674). The SRC values were similar for both groups; 0.011 and 0.009 for control and imidacloprid treated bees, respectively.

## Discussion

Acute imidacloprid exposure at the established field-realistic concentration of 10 ppb has been previously shown to affect flight activity in *B. terrestris* by increasing short-term velocity and decreasing the overall duration and distance flown (15, 25, 26). This study has further shown that exposure to imidacloprid, at a concentration of 10 ppb, increases routine oxygen consumption in the flight muscles of workers. Imidacloprid targets acetylcholine receptors and can result in over-stimulation of the nervous system, initiating an excitatory response (10). We suggest that an increase in oxygen consumption and respiration initiated by this response enables the short-term increase in flight velocity that has been previously reported (15, 27). The enhanced flight velocity in turn may lead to premature exhaustion resulting in a shorter flight duration (15, 28, 29). Hyperactivity caused by neonicotinoids, such as imidacloprid, could also result in long-term muscle exhaustion and impaired energy metabolism as shown with thiamethoxam by Tosi et al. (27).

The higher routine oxygen flux and low spare respiratory capacity detected in the flight muscle of bees exposed to imidacloprid may be further factors contributing to the negative effects on flight activity. The SRC is defined as the mitochondrial capacity to reach additional ATP demands that are greater than routine respiration levels, and to thus prevent ATP crisis (24). This is important in times of stress, exercise, and increased workload when the extra capacity is needed. Hence, the lower SRC in the imidacloprid-exposed bees, which was approximately half of the value for unexposed control bees, suggests a greater mitochondrial dysfunction during times of high ATP demand that is above the routine respiration rate, such as during flight. Imidacloprid increased routine respiration but did not affect the maximum ET capacity; the knock-on effect is likely a diminished SRC. We currently do not know for how long the effects of acute imidacloprid exposure on mitochondria functions last and if the resulting increases in routine respiration are transient. Therefore, the duration of lowered SRC will need to be determined in future follow-up studies.

In the brain, we detected no significant difference in routine and LEAK oxygen consumption between imidacloprid-exposed and control bees, however the maximum ET capacity in imidacloprid-exposed group showed a greater oxygen consumption. This indicates that acute imidacloprid-exposure results in elevated levels of oxygen consumption and thus, in higher maximum respiration rates. However, there was no detected difference in routine or LEAK FCRs, which are likely to be associated with the higher mean routine and LEAK oxygen consumption values. In addition, the FCR values suggest that the routine and LEAK values are in a similar ratio to the maximum ET capacity for both treatment groups. The similarity in the SRC values (0.011 for the control treatment and 0.009 for imidacloprid exposed bees) also illustrates this; these low values indicate that the routine respiration rate in the brain is at maximum ET capacity, as a SRC value of 0.01 represents that the oxygen consumption during routine and maximum ET capacity are equal. Therefore, it appears that imidacloprid increases overall oxygen consumption in the brain; however, due to high inter-individual variance, our results remain inconclusive. This high variability could be associated with the quantity of nAChRs in the brain, which is much greater than in the flight muscle and may therefore explain why there is lower variance among the flight muscles of imidacloprid-exposed bees and among control groups. Cognitive ability such as learning and memory varies among individual bees; this suggests that the brain is particularly susceptible to interspecific variation (30, 31). Hence, a possible variation in the number of neonicotinoid target sites among individual bees may lead to more variability in the response of imidacloprid on mitochondrial function.

Bees are exposed to imidacloprid both acutely and chronically, hence it is important to elucidate the mitochondiral function of both acute and chronic exposure. Chronic exposure of imidacloprid can lead to neuronal dysfunction (13). Neurons require high levels of ATP, and a stable mitochondiral membrane potentital is crucial to sustain ATP production at this level and thereby normal neuronal function (13). Moffat et al. (13) also reported that acute exposure of 10 nM (2.5 ppb) imidacloprid did not reach concentrations in the brain to induce membrane depolarisation. In contrast, 2 day chronic exposure of 1 nM led to an increased sensitivity of bumblebee neurones to acetylcholine, and elicited mitochondrial depolarisaiton of neurons (13). Our findings are in agreement with Nicodemo and colleagues (12), who reported tissue-specific sensitivity to acute imidacloprid exposure in the head and thorax of honeybees and negative effects on ATP synthesis. Our results will help resolve how acute imidacloprid exposure causes a different signature of mitochondiral activity compared to effects of chronic exposure. Moreover, it may now be possible to delineate where acute exposure ends and chronic effects begin.

An aspect of our study is that the worker bees were kept in an enclosed colony and were unable to fly. While this allowed a great deal of experimental control, it should be considered that exercise, such as foraging flights, can increase maximum ET capacity. Hence, mitochondrial function from laboratory-reared non-flying worker bees may vary from that of wild, free-flying and foraging worker bees. Another consideration is that social interactions may also impact brain energetics and mitochondrial function; all bees were kept in small cohorts within small containers from the time of adult eclosion, thus removing them from wider colony interactions and pheromones (7). This may impact brain mitochondrial respiration and behaviour, which may in turn not provide a true representation of *B. terrestris* in a natural or agricultural environment. The effects of acute imidacloprid were only analysed at one time point, shortly after exposure; potential long-term implications of acute exposure on the mitochondrial function in bees remain to be determined. For example, whether mitochondria exposed to imidacloprid return to pre-exposure functioning and, if so, how long such recovery may take.

In this study we have further scrutinised how imidacloprid affects the different mitochondrial respiration states. We found that imidacloprid did not affect LEAK, indicating an increase in oxygen consumption is not associated with proton leak and thus imidacloprid does not seem to have an inhibitory effect on ATP synthase. The increase in routine respiration in the muscle suggests imidacloprid may affect the rate at which electrons flow through the electron transport chain, and thereby increases the rate closer to the maximum ET capacity *via* utilisation of the SRC. A similar mechanism of increased electron transport rate could also explain how imidacloprid increased the maximum ET capacity in the brain. The SRC values were much lower in the brain compared to the flight muscle. This difference between the two tissues could explain how imidacloprid was able to increase the maximum ET capacity in the brain and not the flight muscle as only a slight increase in the rate would be capable of surpassing the maximum ET capacity. The exact mechanism by which imidacloprid affects the electron transport chain requires further investigation.

Overall, we found that acute exposure to imidacloprid, *via* feeding, increased the routine respiration rate in the flight muscle and showed a similar, marginally non-significant, trend towards higher respiration rates in the brain of workers. The higher routine respiration rates in the flight muscle may lead to short-term increases in oxygen consumption and potentially increase ATP production equating to a longer-term deprivation of energy. These results accord with those of previous studies which have shown flight duration and distance to be shorter in bumblebees exposed to imidacloprid. Negative impacts of flight activity will increase exhaustion and mortality of individual bees during flight and this reduced foraging capacity will lead to a reduction in pollination services within natural and agricultural landscapes. The broad range of responses in the brain by individual bees makes it difficult to draw clear conclusions from the measurements. Hence, more research is required to establish the impact of imidacloprid on the brain and elucidate possible explanations for the inconsistencies observed.

These results further contribute to our understanding of how sub-lethal levels of imidacloprid mediate tissue-specific effects on respiration and energy production and thereby impacts pollinator fitness. This study will provide a foundation for further research into the link between mitochondrial function and neonicotinoid exposure, as well as assist in the development and assessment of novel pesticides that minimise harmful effects on non-target species.

## Data Availability Statement

The original contributions presented in the study are included in the article/Supplementary Material, further inquiries can be directed to the corresponding author.

## Author Contributions

CS and RS designed the experiment with the additional support of LC and BE. Bee husbandry, laboratory work, and data collection were performed by CS. HRR was performed by CS with the support of LC and BE. Data analysis was performed by CS with supplementary analysis by CS and IH. Figures were prepared by CS. Paper was written by CS and RS with additional editorial assistance by LC, BE, IH, and TD. All authors contributed to the article and approved the submitted version.

## Funding

CS is a PhD candidate funded by the Future Food Beacon/Graduate Centre for International Agriculture, University of Nottingham and Rothamsted Research and BE is a PhD candidate funded by the University of Nottingham Biotechnology and Biological Sciences Doctoral Training Programme (BBSRC-DTP).

## Conflict of Interest

The authors declare that the research was conducted in the absence of any commercial or financial relationships that could be construed as a potential conflict of interest.

## Publisher's Note

All claims expressed in this article are solely those of the authors and do not necessarily represent those of their affiliated organizations, or those of the publisher, the editors and the reviewers. Any product that may be evaluated in this article, or claim that may be made by its manufacturer, is not guaranteed or endorsed by the publisher.
