# Corrigendum: Acute imidacloprid exposure alters mitochondrial function in bumblebee flight muscle and brain

**DOI:** 10.3389/finsc.2024.1415939

**Published:** 2024-04-22

**Authors:** Chloe Sargent, Brad Ebanks, Ian C. W. Hardy, T. G. Emyr Davies, Lisa Chakrabarti, Reinhard Stöger

**Affiliations:** ^1^ School of Biosciences, Sutton Bonington Campus, University of Nottingham, Loughborough, United Kingdom; ^2^ School of Veterinary Medicine and Science, Sutton Bonington Campus, University of Nottingham, Loughborough, United Kingdom; ^3^ Department of Agricultural Sciences, University of Helsinki, Helsinki, Finland; ^4^ Department of Biointeractions and Crop Protection, Rothamsted Research, Harpenden, United Kingdom; ^5^ Medical Research Council Versus Arthritis Centre for Musculoskeletal Ageing Research, Birmingham, United Kingdom

**Keywords:** *Bombus terrestris*, mitochondria, imidacloprid, oxidative phosphorylation, high-resolution respirometry

In the published article, there was an error in **Figure 1** as published. The LEAK respiration was incorrectly labelled. The corrected **Figure 1** and its caption appear below.

**Figure 1:**
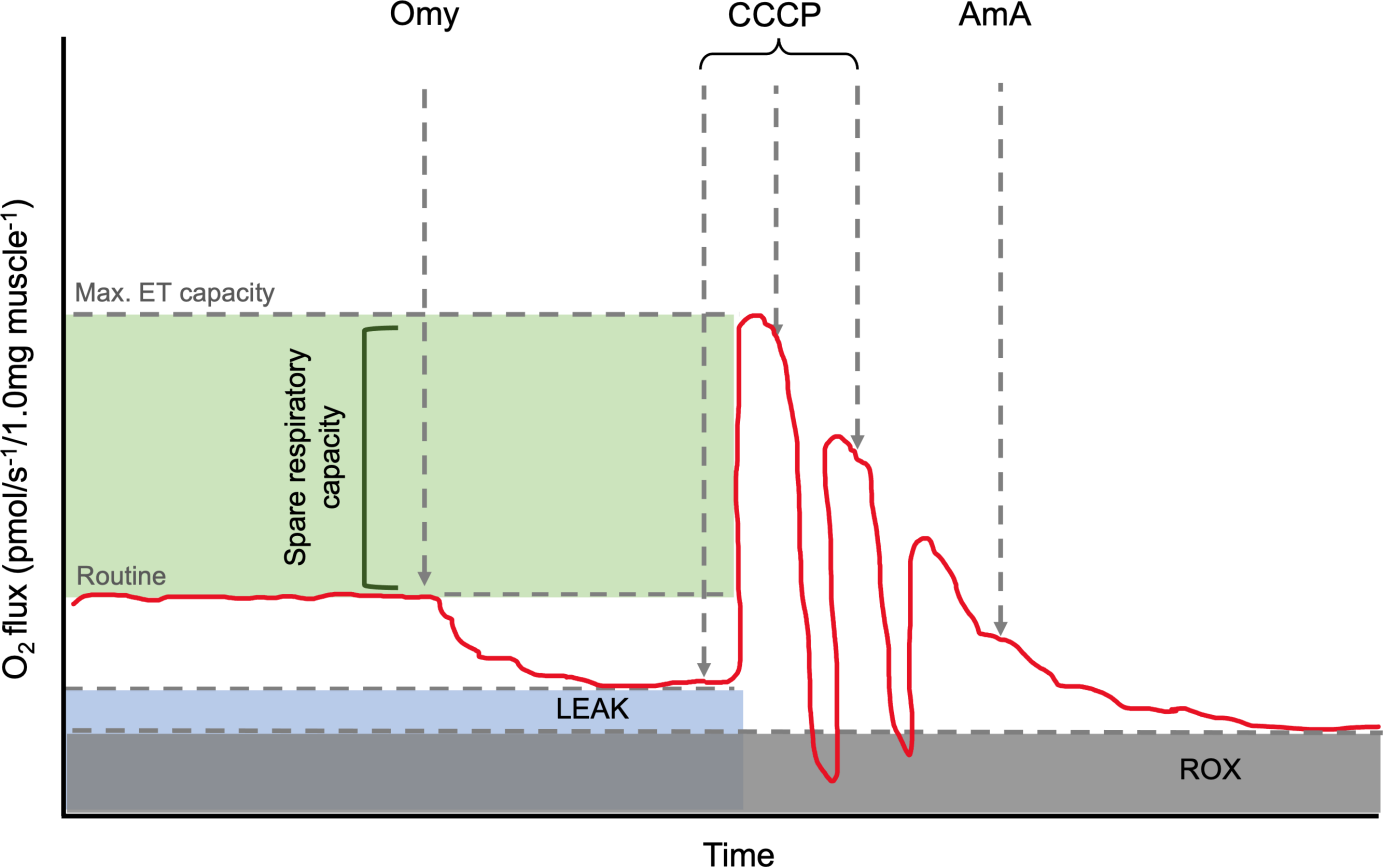
Annotated representation of the O2k oxygraph output. The red line is the oxygen flux. Routine is shown before the addition of Omy. LEAK state can be determined after the inhibition of ATP synthase by Omy. The maximum electron capacity is determined as the highest peak after CCCP titrations. The spare respiratory capacity and its relative value can be calculated *via* the calculation, [(ET capacity specific flux)/(Routine specific flux × 100)]. AmA, a complex III inhibitor, is added to determine the residual oxygen consumption (ROX) as the baseline state and allow for background correction. Omy, oligomycin; CCCP, carbonyl cyanide m-chlorophenyl hydrazone; AmA, antimycin A.

The authors apologize for this error and state that this does not change the scientific conclusions of the article in any way. The original article has been updated.

