# Supplementary material for: Acute Imidacloprid Exposure Alters Mitochondrial Function in Bumblebee Flight Muscle and Brain
Source: Front Insect Sci. 2021 Dec 1;1:765179. doi: 10.3389/finsc.2021.765179 (PMC10926543; doi:10.3389/finsc.2021.765179)
Supplement: Supplementary file 1 [file Table_1.DOCX]

**Supplementary Materials**

**Acute imidacloprid exposure alters mitochondrial function in bumblebee flight muscle and brain**

Chloe Sargent^1*^, Brad Ebanks^2*^, Ian C.W. Hardy^1,2,3^, T.G. Emyr Davies^4^, Lisa Chakrabarti^2,5^, Reinhard Stöger^1^

^1^School of Biosciences, University of Nottingham, Sutton Bonington Campus, United Kingdom

^2^School of Veterinary Medicine and Science, University of Nottingham, Sutton Bonington Campus, United Kingdom

^3^Department of Agricultural Sciences, University of Helsinki, Finland

^4^Rothamsted Research, Harpenden, United Kingdom

^5^MRC Versus Arthritis Centre for Musculoskeletal Ageing Research, Birmingham, UK

**Controlling for multiple comparisons**

We tested for effects on brain and muscle tissue using 10 closely related statistical hypothesis tests on data from the same set of bees. Here we use the false discovery rate (FDR) procedure to control for Type I erros (Benjamini & Hochberg, 1995; McDonald, 2014). We treated results presented in Table 1 of the main paper as family of tests and set the family-wide α-value to a relatively stringent 0.10, which may in consequence generate Type II errors. The intially significant result for an effect of imidocloprid on flight muscle FRC was no longer significant but the other two signfiicant results from separate analyses retained their significance (**Table S1**).

**Table S1. Multiple comparisons testing of results reported in the main paper.** Results are reported in order of increasing P-value. FDR was set to 0.10.

| **Tissue and measure tested** | **P-values**  (from Table 1) | **Benjamini-Hochberg significance** |
| --- | --- | --- |
| Fight muscle Routine Oxygen flux | 0.012 | Significant |
| Brain ET Capacity Oxygen flux | 0.017 | Significant |
| Flight muscle Routine FCR | 0.047 | Not significant |
| Brain Routine Oxygen flux | 0.06 | Not significant |
| Brain LEAK Oxygen flux | 0.097 | Not significant |
| Flight muscle ET Capacity Oxygen flux | 0.136 | Not significant |
| Flight muscle LEAK FCR | 0.196 | Not significant |
| Flight muscle LEAK Oxygen flux | 0.376 | Not significant |
| Brain Routine FCR | 0.639 | Not significant |
| Brain Leak FCR | 0.67 | Not significant |

**Analysis of the morphology of bees and external factors on HRR results in each treatment group.**

**Table S2. Mean ages, sizes, and sucrose consumed before high-resolution respirometry (HRR) analysis in worker bumble bees.** Intertegular distance (ITD) was used as a measure of body size. Unpaired Student’s t-tests found no significant difference in mean age (flight muscle: *t*_df=6_ = -0.42, *p* = 0.691; brain: *t*_df=12_ = -0.47, *p* = 0.644), ITD (flight muscle: *t*_df=6_ = 0.41, *p* = 0.696; brain: *t*_df=12_ = -0.21, *p* = 0.840) or the amount of sucrose consumed (flight muscle: *t*_df=6_ = 0.18, *p* = 0.866; brain: *t*_df=12_ = -1.1, *p* = 0.278) between treatment groups for both tissues.

|  | **Flight muscle** | | **Brain** | |
| --- | --- | --- | --- | --- |
|  | Control  n = 4 | IMD  n = 4 | Control  n = 7 | IMD  n = 7 |
| Age (days)  Mean  Range | 16  14-20 | 17  15-20 | 11  5-14 | 11  10-13 |
| Mean ITD (mm) | 5.87 | 5.76 | 5.50 | 5.56 |
| Mean sucrose consumed (mg) | 81.9 | 77.0 | 48.0 | 59.5 |

**Table S3**: **Correlation between sucrose consumption and oxygen consumption at three respiration states.** Pearson’s correlation coefficient was calculated to determine association between the amount of sucrose consumed and oxygen flux and different respiration states.

|  | Flight Muscle | | | Brain | | |
| --- | --- | --- | --- | --- | --- | --- |
|  |  | *p* | cor |  | *p* | cor |
| Routine | *t*_df=6_ = -0.446 | 0.671 | -0.179 | *t*_df=12_ = -0.58 | 0.572 | -0.165 |
| LEAK | *t*_df=6_ = -0.135 | 0.897 | -0.055 | *t*_df=12_ = -0.48 | 0.639 | -0.138 |
| ET capacity | *t*_df=6_ = 2.18 | 0.072 | 0.666 | *t*_df=12_ = -0.26 | 0.800 | -0.075 |

**References**

Benjamini, Y. & Hochberg, Y. (1995). Controlling the false discovery rate: a practical and powerful approach to multiple testing. J R Statist Soc B*.* 57: 289-300.

McDonald, J. (2014). Handbook of Biological Statistics. Baltimore, MD: Sparky House Publishing.
